# Oscillating Positive Airway Pressure Versus CPAP for the Treatment of Obstructive Sleep Apnea

**DOI:** 10.3389/fmed.2015.00029

**Published:** 2015-05-11

**Authors:** José Haba-Rubio, Nicolas Julien Petitpierre, Françoise Cornette, Nadia Tobback, Sopharat Vat, Theresia Giallourou, Ahmed Al-Jumaily, Raphael Heinzer

**Affiliations:** ^1^Center for Investigation and Research in Sleep, University Hospital of Lausanne (CHUV), Lausanne, Switzerland; ^2^Laboratory of Hemodynamics and Cardiovascular Technology, Swiss Federal Institute of Technology, Lausanne, Switzerland; ^3^Institute of Biomedical Technologies, Auckland University of Technology, Auckland, New Zealand

**Keywords:** pharynx, vibration, arousal, obstructive sleep apnea, continuous positive airway pressure

## Abstract

Although continuous positive airway pressure (CPAP) is the most effective therapy for obstructive sleep apnea (OSA), it is not always well tolerated by the patients. Previous physiological studies showed that pressure oscillations applied to the pharynx could activate upper airway muscles, but it is not clear whether these pressure oscillations could be tolerated during sleep in OSA patients. The aim of this study was to assess the tolerance of oscillating positive airway pressure (O-PAP) (a CPAP device delivering high-frequency pressure oscillations to the upper airway) compared to CPAP. Fourteen OSA patients currently on CPAP [age 59.9 ± 10.1 years old, BMI 34.8 ± 7.2 kg/m^2^, initial apnea–hypopnea index (AHI): 58.7 ± 25.2 events/h] used O-PAP or CPAP on two consecutive nights under polysomnography, in a single-blind randomized crossover design to assess sleep quality. A subtherapeutic pressure (70% of the optimal titrated pressure) was applied in both conditions and the residual AHI with each technique was also compared. There was no difference in measured or perceived sleep quality between the two treatment modalities (sleep efficiency 90.0% versus 88.1%, *p* = 0.54). Despite the small sample, we also found a trend toward a decrease in residual respiratory events with O-PAP compared to CPAP (median AHI 14.3 versus 20.5/h, *p* = 0.194). The good tolerance of O-PAP and the positive trend toward a reduction in residual AHI should stimulate further research on the effects of O-PAP in OSA patients.

## Introduction

Obstructive sleep apnea (OSA) syndrome is caused by repetitive closure of the pharynx during sleep. Although continuous positive airway pressure (CPAP) has been accepted as the standard treatment for moderate to severe OSA (1), many patients report discomfort or cannot tolerate it. Some of the common side effects such as air leaks, mask discomfort, or aerophagia are related to the pressure level applied (2). Therefore, any means allowing a reduction of the pressure level without jeopardizing the primary CPAP function might improve patients’ comfort and, possibly, treatment adherence.

The collapse of the upper airway in OSA is mainly due to a failure of the pharyngeal dilator muscles to counteract the negative intraluminal pressure generated by the inspiratory muscles. During wakefulness, the pharynx is kept opened by tonic and phasic activations of the dilator muscles during the breathing cycle. These muscles are also activated by a reflex (the “pharyngeal dilators reflex”) triggered by mechanoreceptors located in the pharynx and larynx, responding to intraluminal negative pressure. In OSA patients, many abnormalities have been described in the neuromuscular control of the upper airway, including histological evidence of denervation, decreased sensitivity to vibrations and temperature, and EMG evidence of motor neuropathy (3).

Therefore, dilator muscles are an interesting target in OSA treatment and studies investigating oropharyngeal muscle training by exercises (4), or direct stimulation (5–7), have shown a positive impact on respiratory events.

In an animal study, Plowman et al. demonstrated that high-frequency–low-pressure oscillations delivered to the upper airway of dogs could trigger an augmentation of the genioglossus (GG) electromyographic activity, both during wakefulness and sleep (8). In a human physiological study, Henke and Sullivan showed that intermittent high-frequency–low-amplitude pressure oscillations also had a stimulating effect on the upper airway muscle tone (9). When applied to single breaths in sleeping subjects, this oscillating pressure induced a significant activation of the GG muscle and an increase in tidal volume in controls and OSA patients.

Based on these physiological studies, an experimental device was developed (the oscillating positive airway pressure, or “O-PAP”) to deliver pressure oscillations superimposed onto CPAP therapy. As it was unclear whether these pressure oscillations could be tolerated during sleep, we designed this study to assess sleep quality under O-PAP, compared to CPAP.

## Subjects and Methods

### Participants

Subjects were recruited among CPAP-treated OSA outpatients followed at the Center for Investigation and Research in Sleep (Lausanne, Switzerland). Inclusion criteria were >18 years of age, initial apnea–hypopnea index (AHI) >15/h, good tolerance to CPAP, mean use of CPAP of >4 h/night in the last 3 months, and residual AHI on CPAP <10/h. Exclusion criteria were sleep-related disorders other than OSA, any other disorder disturbing sleep, use of opiates, benzodiazepines, and muscle relaxants, unstable cardiovascular disease, and inability to consent.

### Design and set-up

All participants underwent two supervised full polysomnographies, one with CPAP only and one with O-PAP. They were randomized in blocks of four to receive CPAP or O-PAP first. During the first night, a CPAP titration was performed, starting in the first NREM2 period, using an initial pressure of 4 cmH_2_O and increasing by steps of 1 cmH_2_O until control of apneas and hypopneas was achieved. Seventy percent of this optimal pressure was then applied during both polysomnographies. The titration time was not included in the analyses.

**Figure 1:**
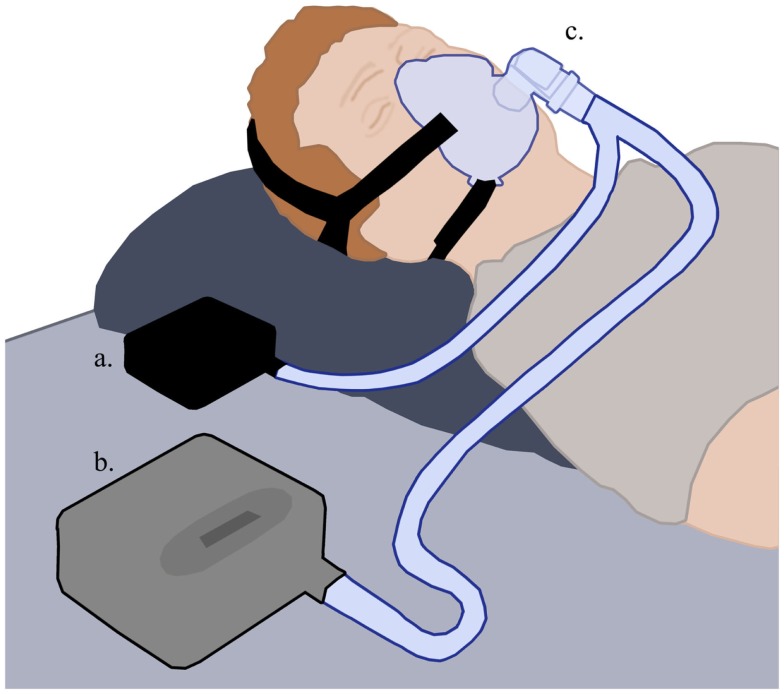
**The O-PAP setting**. The pressure oscillation generator (a) and the CPAP (b) are connected to the patient’s mask through a “Y” tubing (c). Modified from Wikimedia Commons.

**Figure 2:**
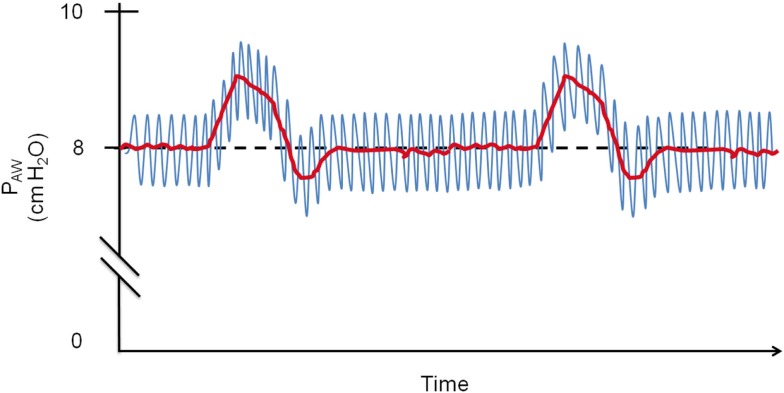
**Schematic drawing of O-PAP in a patient with fixed 8 cmH_2_O CPAP: the O-PAP produces 1 cmH_2_O pressure oscillations (blue line) that are superimposed onto the pressure generated by the CPAP (red line)**. PAW, airway pressure.

Pressure oscillations were administered using a custom-made pressure oscillator connected to the CPAP through a “Y” tubing (Figures 1 and 2). The oscillations’ amplitude was fixed (1 cmH_2_O) and the frequency was titrated by the technician while the patient was awake, starting with 15 Hz, until the patient perceived the vibrations in his throat and not his mouth or thorax.

The polysomnographies were performed using an Embla N7000™ and analyzed with the Somnologica™ software (Embla Systems, Inc., Broomfield, CO, USA). One investigator blinded to treatment modality scored sleep stages according to the American Academy of Sleep Medicine (AASM) 2007 recommendations (10), and respiratory events using the criteria from the AASM update published in 2012 (11).

The main study endpoints were total sleep time, arousals index, respiratory arousals index, sleep efficiency, and the Leeds sleep evaluation questionnaire (LSEQ) (12). The secondary endpoints were the residual AHI under a suboptimal pressure (70% of the optimal titrated pressure), the AHI according to the body position and the sleep stages, and the oxygen desaturation index (ODI) ≥3%.

### Statistics

Sample size was estimated empirically, as there was no data available on the effect of O-PAP on sleep quality. We estimated that we needed to include 12 patients to have an 80% power to detect a difference of 5% in sleep efficiency with a SD of 6% (1 − β = 0.8, α = 0.05). Statistical analysis was made using Medcalc statistical software (MedCalc Software, 8400, Ostend, Belgium). Results are reported as mean ± SD, or median ± interquartile range (IQR). To compare different outcomes between CPAP and O-PAP, we used paired *t*-tests for normally distributed data and Wilcoxon tests for non-normally distributed data.

The study was approved by the local ethics committee (protocol 296/12) and all patients gave written informed consent.

## Results

Fourteen subjects were included in the study. Their characteristics are shown in Table 1. Seventy-one percent of them were obese (BMI >30 kg/m^2^) and all but one had severe OSA (AHI >30/h). The median pressure applied during both nights (~70% of titrated pressure) was 6.0 [4.3–9.8] cmH_2_O. The mean oscillation frequency applied was 27.1 ± 6.9 Hz.

**Table 1:** **Patients’ characteristics**.

	Mean	SD
Age (years)	59.9	±10.1
BMI (kg/m^2^)	34.8	±7.2
Sex M/F (*N*)	13/1	
Neck circumference (cm)	45.0	±4.4
Initial AHI (events/h)	58.7	±25.2
CPAP use (years)	4.1	±1.8
CPAP pressure^a^ (cmH_2_O)	12.3	±2.9
Type of mask (N/FF)	6/8	
aCPAP/CPAP, N	12/2	

*^a^Fixed pressure or pressure at 95th percentile for aCPAP*.

*N, nasal; FF, full face; aCPAP, autoadjusted CPAP*.

Comparison of the respiratory and sleep parameters under both conditions is shown in Table 2. Sleep quality parameters (arousal index, respiratory arousals index, sleep time, and sleep efficiency) and the perceived sleep quality assessed by the LSEQ were not altered by O-PAP. There was a trend toward a decrease in AHI and ODI with O-PAP compared to CPAP alone, especially in NREM sleep and in the supine position, but none of these differences reached statistical significance.

**Table 2:** **Objective and subjective sleep quality parameters and residual AHI with O-PAP and CPAP under a pressure corresponding to 70% of the optimal CPAP pressure**.

	CPAP	O-PAP	*p*
Arousal index (/h)	32.5 [23.7–44.4]	28.1 [22.5–34.7]	0.296
Respiratory arousals index (/h)	16.2 [11.0–21.2]	11.5 [5.4–20.8]	0.104
Sleep time^a^ (min)	356.6 [232.8–390.2]	336.6 [234.5–361.0]	0.325
Sleep efficiency (%)	90.0 [78.0–92.5]	88.1 [71.9–92.6]	0.542
Sleep quality VAS (mm)	78.8 [60.6–85.7]	75.5 [57.4–85.4]	0.695
AHI (/h)	20.5 [13.7–28.9]	14.3 [8.5–25.8]	0.194
AHI supine (/h)	36.9 [24.0–58.4]	23.6 [11.5–66.8]	0.432
AHI non-supine (/h)	11.7 [4.5–16.9]	12.3 [4.4–21.9]	0.966
AHI in REM sleep (/h)	7.9 [5.2–13.6]	12.6 [9.1–26.7]	0.426
AHI in NREM sleep (/h)	22.4 [13.7–30.9]	13.2 [8.2–22.2]	0.104
ODI ≥3% (/h)	16.2 [9.9–35.6]	12.7 [9.4–22.1]	0.583

*^a^Sleep time does not include titration*.

*REM, rapid eye movement sleep; NREM, non-rapid eye movement sleep; VAS, visual analog scale*.

*Maximum score 100 mm. Higher score indicates better sleep quality*.

*Results are expressed as median and interquartile range*.

## Discussion

The main finding of this study is that O-PAP is well tolerated and does not alter objective nor subjective sleep quality in OSA patients. This suggests that the 1 cmH_2_O pressure oscillations applied with O-PAP do not disturb sleep, even when used during the whole night in OSA patients.

As shown by Plowman et al. (8), pressure oscillations delivered to the upper airway of dogs can trigger an increase in GG EMG activity. In a human physiological study, Henke and Sullivan showed that intermittent pressure oscillations also had a stimulating effect on the upper airway muscle tone (9). It is thus possible that the constant oscillations generated by the O-PAP could stimulate GG activity and decrease the CPAP pressure required to prevent upper airway collapse and improve the patient’s tolerance to the treatment. Even though our sample was not large enough to determine the effect of continuous oscillations on the AHI, we found an interesting trend toward a decrease in the residual AHI compared to standard CPAP under subtherapeutic pressure. These preliminary results should stimulate further research on the effects of O-PAP in OSA patients.

In our study, O-PAP seems to have a greater effect in the supine position and in NREM sleep but not in non-supine position and in REM sleep. This may be due to an altered GG responsiveness since a lower GG activation in response to negative pharyngeal pressure was previously reported in non-supine positions (13) and in REM sleep (14). We can also anticipate that O-PAP could be more efficient in younger patients with preserved pharyngeal sensitivity to air vibration since long-term snoring and sleep apnea are associated with altered pharyngeal sensitivity (15), probably due to sensitive nerve injuries. To determine whether O-PAP could allow treating OSA with lower pressures than CPAP, larger studies including pharyngeal sensitivity measurements and fine CPAP/O-PAP titrations are required.

Our study has a few limitations. First, due to the easily perceived O-PAP vibrations, patients or PSG technician blinding was not feasible. However, the investigator interpreting the polysomnographies was blinded to treatment allocation. Second, our sample size was small. Finally, because the study was powered to find a difference in sleep efficiency, it did not have enough power to detect a significant effect difference between O-PAP and regular CPAP on AHI.

In conclusion, O-PAP seems to be a safe and well-tolerated therapy that does not affect subjective nor objective sleep quality. The positive trend we found toward a reduction in residual AHI with O-PAP should stimulate further research with larger groups of patients to better determine if O-PAP could control OSA with lower pressure than standard CPAP.

## Author Contributions

RH: design of the study, methodology, data analysis, manuscript writing. NP: data analysis, manuscript writing. FC, NT, and SP: data collection and analysis, manuscript review. TG: conception, methodology, manuscript review. AA-J: design of the study, inventor of the O-PAP device, manuscript review. JH-R: design of the study, data collection, manuscript writing. All the authors vouch for the accuracy and completeness of the report. All the authors approved the final draft.

## Conflict of Interest Statement

The authors report no conflict of interest in relation with the present study. The O-PAP device is under no patent.
